# Brain size is reduced by selection for tameness in Red Junglefowl– correlated effects in vital organs

**DOI:** 10.1038/s41598-017-03236-4

**Published:** 2017-06-12

**Authors:** Beatrix Agnvall, Johan Bélteky, Per Jensen

**Affiliations:** IFM Biology, Avian Behavioural Genomics and Physiology group, Linköping University, 581 83 Linköping, Sweden

## Abstract

During domestication animals have undergone changes in size of brain and other vital organs. We hypothesize that this could be a correlated effect to increased tameness. Red Junglefowl (ancestors of domestic chickens) were selected for divergent levels of fear of humans for five generations. The parental (P0) and the fifth selected generation (S5) were culled when 48–54 weeks old and the brains were weighed before being divided into telencephalon, cerebellum, mid brain and optic lobes. Each single brain part as well as the liver, spleen, heart and testicles were also weighed. Brains of S5 birds with high fear scores (S5 high) were heavier both in absolute terms and when corrected for body weight. The relative weight of telencephalon (% of brain weight) was significantly higher in S5 high and relative weight of cerebellum was lower. Heart, liver, testes and spleen were all relatively heavier (% of body weight) in S5 high. Hence, selection for tameness has changed the size of the brain and other vital organs in this population and may have driven the domesticated phenotype as a correlated response.

## Introduction

Domestication has dramatically altered the phenotypes of animals due to selection on different traits chosen by humans. Examples of common morphological effects seen in many different species are changes in body size^1^, body proportions^2^ and brain size^3^. It has been suggested that the occurrence of common phenotypes in domesticated animals might be a result of correlated responses to selection for one or a few traits, for example mediated by pleiotropy or linkage^4, 5^. This hypothesis is largely supported by the famous farm-fox project, where silver foxes developed several dog-like traits after only few generations of selection for tameness only^5^. Since the common denominator for any successful domestication of animals is an initial reduction in fear of humans, the evolution of domesticated phenotypes may be driven by selection for tameness.

We have investigated this idea by selection of Red Junglefowl (*Gallus gallus*) for increased tameness, and measuring possible correlated effects. This species is considered the ancestor of all modern chickens, and domestication started in South East Asia about 8000 years ago^6, 7^. Like all other domesticates, chickens differ from their wild ancestors in a number of traits, such as reproduction capacity^8^ growth^9^, colouration^10^, organ size (brain, intestines)^11^ and in behaviour such as fear towards humans^12^, exploration and foraging^13^. The effects of domestication on the chicken are therefore well investigated but it still remains an open question whether this phenotypic complex is driven by tameness.

We have previously reported that selection for tameness in Red Junglefowl leads to correlated responses in a range of behavioural and physiological traits after only a few generations^4, 14–16^. Starting from an outbred population, we found a significant genetic component underlying fear of humans, and the trait is genetically correlated to other behaviors such as exploration^15^. After only three generations, animals selected for low fear of humans grew larger, laid larger eggs and generated larger offspring. Low-fear animals also performed more aggressive behaviours during a social dominance test. Overall, animals selected on low fear of humans appear to be more adapted to the housing environment in which they were selected^14^. Furthermore, in the fifth generation basal metabolic rate, feed efficiency and boldness in a novel object test was higher in the animals selected on low fear of humans^4^. In the fifth selected generation, gene expression in thalamus/hypothalamus differed for at least 33 genes, mainly related to sperm and immunological functions^16^.

As mentioned earlier, one part of the domesticated phenotype concerns alterations of morphological traits and organ sizes. One commonly observed effect is a reduction in overall brain size. This could partly be caused by the difference in life style between domesticated animals and their ancestors in terms of challenges to survive and reproduce^17^. The brain is a highly energy demanding organ and may therefore be reduced as a trade-off with other physiological traits, such as reproduction and growth^18^. Also other organs change during the domestication process, and in chickens, fast growing breeds for meat production have relatively larger intestines and smaller brains and leg bones^11^.

In this paper we investigate the morphological differences in brain and some other internal organs between Red Junglefowl selected for either high or low fear of human. Our hypothesis is that selection for tameness may drive alterations in sizes of vital organs by means of correlated responses.

## Results

**Figure 1:**
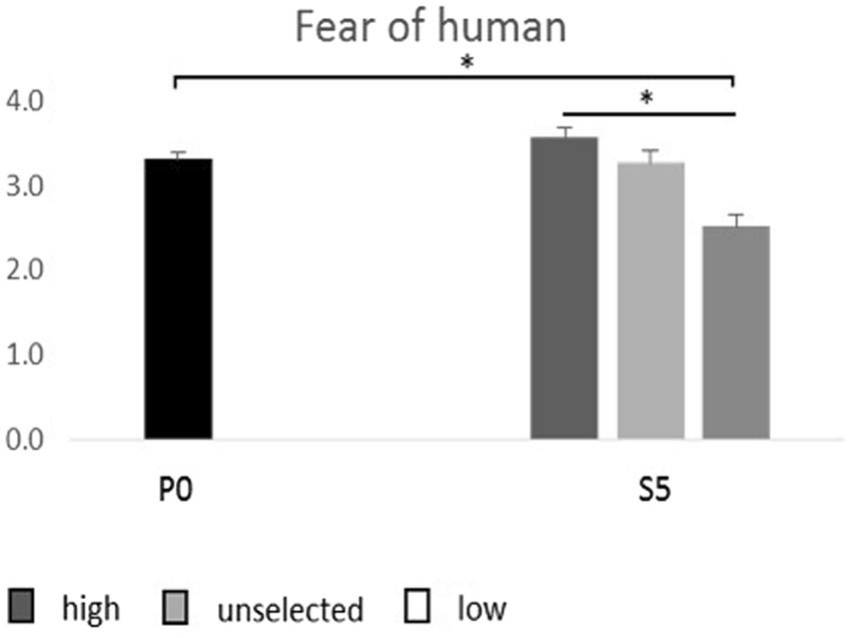
Average fear scores (+/− SEM) in the parental generation (P0) and in the three selection lines in the fifth selected generation (S5). The fear score was obtained through transformation of behavioural recordings in a fear-of-human-test to a scale ranging from 1–5, where 1 signifies the least fearful reactions. *P < 0.05.

In the fear-of-human test conducted on S5 birds, S5 high (selected for high fear of humans) had the highest score, S5 unselected (not intentionally selected) intermediate and S5 low (selected for low fear of humans) the lowest (F_2.106_ = 8.98; P < 0.001) (Fig. 1). S5 low also had significantly lower score than the average P0 score (F_1.92_ = 19.45; P < 0.001), and there was a tendency for S5 high to have higher scores than the average P0 score (F_1.98_ = 3.01; P = 0.08). There was no difference between the scores of S5 unselected and the average P0 score.

**Figure 2:**
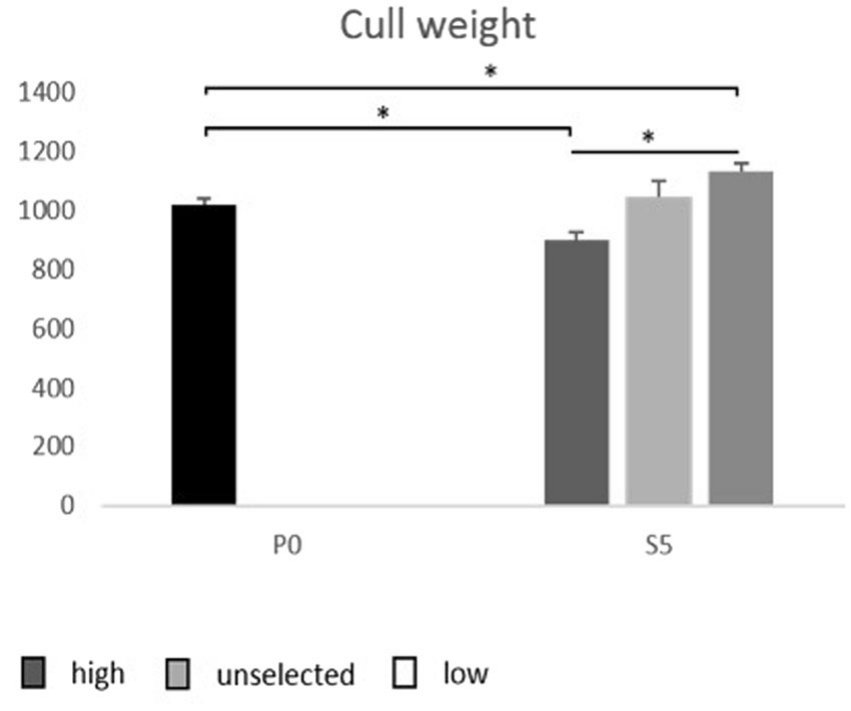
Average weights at culling in g (+/− SEM) in the parental generation (P0) and the three selection lines in the fifth selected generation (S5). *P < 0.05

S5 High had a significantly lower body weight than the average of P0 (F_1.85_ = 19.51; P < 0.001), and S5 Low weighed significantly more than the average of P0 (F_1.8_ = 4.87; P < 0.03) (Fig. 2). Within S5 there was a significant effect of selection on body weight (F_2.58_ = 28.72; P < 0.001), with S5 low being the heaviest and S5 high the lightest. Within S5 there was also a significant interaction between selection and sex where males were larger than females in all selection lines, but this was most pronounced in S5 Low and S5 unselected (F_2.58_ = 4.77; P < 0.012), indicating that selection for tameness affected male and female body masses to different degree. The sex differences in body weight as well as for different brain regions, as explained below, are further elaborated in the Supplementary Figure S1.

**Figure 3:**
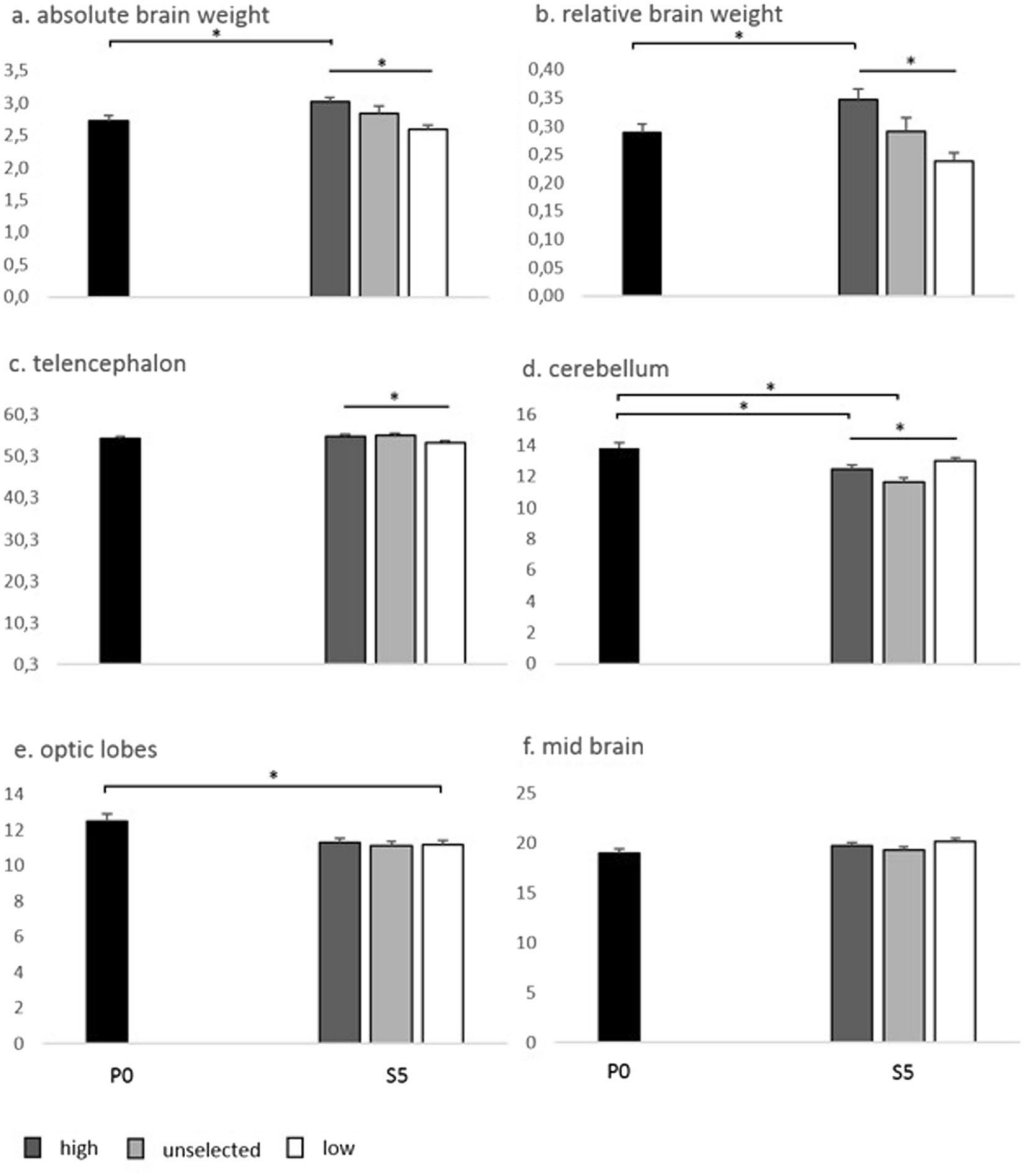
Average brain weights (+/− SEM) in the parental generation (P0) and the three selection lines in the fifth selected generation (S5). (**a**) Absolute weight in g of the brain; (**b**) Relative weight of the brain in % of body weight; (**c**–**f**); Relative weight of different brain parts in % of total brain weight. *P < 0.05.

The total brain weight, uncorrected for body weight, differed significantly between selection lines within S5, where S5 High had the largest brains (F_2.58_ = 10.19; P < 0.001). Total brain weight, uncorrected for body weight, also differed significantly between P0 and S5 High (F_1.84_ = 10.38; P < 0.002) (Fig. 3a and b), whereas there were no significant differences between P0 and the other two selection lines in this respect. With respect to the relative brain weight (corrected for body size), also in this case S5 High had significantly larger brains when comparing the selection lines within S5 (F_2.58_ = 15.75; P < 0.001) and between P0 and S5 High (F_1.85_ = 8.44; P < 0.005). Furthermore, there was a significant difference between the sexes (irrespective of selection) within S5 on the relative brain weight where females had relatively larger brains (F_1.58_ = 71.586; P < 0.001)

Focusing on the relative sizes of different parts of the brain (in proportion of the entire brain), S5 high had significantly larger telencephalon than S5 low (F_2.58_ = 4.07; P < 0.03) but there were no significant differences between the average of P0 and any of the selection lines in S5 (Fig. 3c). There was an effect of sex within S5 where females had relatively larger telencephalon than males (F_1.58_ = 5.071; P = 0.028).

S5 low had significantly higher relative cerebellum weight than S5 unselected and S5 high (F_2.58_ = 6.24; P < 0.003). Both S5 high and S5 unselected had smaller relative cerebellum than the average of P0 (Fig. 3d). In S5, males had larger cerebellum than females (F_1.58_ = 6.384; P = 0.014).

There were no significant differences in the relative weight of the optic lobes within S5 (F _2.58_ = 0.12; P < 0.9). However, S5 low had significantly smaller optic lobes than the average of P0 (F_1.85_ = 4.23; P < 0.043) (Fig. 3e).

The relative weight of the midbrain did not differ between selection lines within S5, or between the average of P0 and any of the selection lines within S5, or between the sexes (Fig. 3f).

The relative sizes of heart, liver, spleen and testis in each selection line is summarized in Table 1. Within S5, S5 high animals had relatively larger hearts (corrected for body weight) than S5 unselected and S5 low animals (F_2.58_ = 9.47; P < 0.001). They also had significantly larger relative heart size than the average P0 (F_1.85_ = 18.72; P < 0.001). Males had significantly larger relative heart size than females in S5, (F_1.58_ = 6.645; P = 0.012).

**Table 1:** Mean organ weight in percent of body weight (+/− SEM) in the parental generation (P0) and the fifth generation (S5) selected for “High” or “Low” fear of humans, or unselected.

Ratio organ/body weight	P0			S5								
				High			Unselected			Low		
	Mean	SEM		Mean	SEM		Mean	SEM		Mean	SEM	
Heart	0.398	±	0.010	0.483		0.019	0.395		0.018	0.379	±	0.022
Total testis	0.590	±	0.043	0.72±2	±	0.036	0.471	±	0.076	0.654	±	0.033
Proportion left/right testis	0.836	±	0.021	1.321	±	0.045	1.272	±	0.090	1.238	±	0.037
Liver	1.963	±	0.117	1.958	±	0.179	1.757	±	0.211	1.432	±	0.129
Spleen	0.085	±	0.003	0.115	±	0.006	0.085	±	0.005	0.076	±	0.007

S5 high had larger relative liver sizes than both S5 unselected and S5 low (F_2.57_ = 4.41; P < 0.016). There were no differences in liver size between the average P0 and any of the selection lines in S5, but there was a sex effect within S5, with females having significantly larger relative liver sizes than males (F_1.57_ = 239.28; P < 0.001).

S5 high had the largest relative spleen size (F_2.58_ = 9.39; P < 0.003). S5 high also had significantly larger spleens than the average P0 (F_1.85_ = 27.58; P < 0.001).

S5 high had significantly larger total testis weight than the other selection lines (F_2.32_ = 6.92; P < 0.003). The proportion of right vs left testis did not differ between the selection lines within S5 but the average P0 proportion was significantly smaller than all the three lines of S5 (P0-S5 High F_1.40_ = 126.1; P < 0.001; P0-S5 Unselected F_1.36_ = 52.69; P < 0.001; P0-S5 Low F_1.43_ = 106.33; P < 0.001).

## Discussion

Our findings show that the absolute and relative sizes of major organs, notably the brain, can change as a result of correlated responses to selection for tameness in Red Junglefowl. This indicates that domesticated phenotypes may to some extent evolve as a correlated and secondary effect of reduced fear of humans, a trait that is of central relevance for the initial phases of domestication. Furthermore, our results show that modifications of brain size may selectively act on only some brain regions, indicating that the correlated effects may possibly be adaptive, affecting specific brain functions.

Domestication is a powerful model for evolution, where the selective pressures acting on the population are relatively well known. A common set of phenotypic changes is associated with domestication in many species, including behavioural, physiological and morphological alterations^19^. This is often referred to as the domesticated phenotype, and it remains unknown whether it is the result of repeated independent selection of each trait by humans, or whether it is a result of correlated responses to a common selection factor. It has been suggested that tameness may be this driving factor, since reduced fear of humans appears to be crucial for the successful domestication of any animal species^4, 5, 20^. We have previously demonstrated, using the same Red Junglefowl population as in the present paper, that selection on reduced fear of humans simultaneously affects social behaviour, growth, reproduction and metabolism^4, 14, 15^. In the present study, we investigated whether also the changes in organ size and morphology commonly associated with the domesticated phenotype, primarily the reduction in brain size seen in most domesticates^21^, might occur secondarily to increased tameness.

The animals used in this study were selected for high or low fear of humans in a standardized test for five generations. As expected, this caused a clear a difference between parental birds (P0) and the selected ones in the fifth generation (S5). Notably, the unselected birds did not differ significantly from P0, suggesting that genetic drift has not been a major factor in the results observed here. Furthermore, there were few significant differences between the weights of different brain parts when comparing P0 and S5 low, so the main effects appear to have occurred in the birds selected for higher fear levels.

Both the relative and absolute brain weight differed between selection lines, with high fear birds having the heaviest. Although the differences in S5 were mainly caused by an increased brain size in the most fearful animal relative to the parental birds, showing that effects of selection for tameness affected brain size, and possibly brain function, in a complex way. However, although not possible to test with our present data, the significantly smaller brains in the low fear-birds is consistent with the brain regression hypothesis^22^. According to this hypothesis, domesticated (and therefore less fearful) animals adaptively reduce the brain size in order to reallocate energy and resources to traits such as reproduction. This is also central to the resource allocation theory^23^ which states that animals adaptively allocate resources to different biological functions in relation to the demands of their specific ecological niche, which in the case of domesticates would be a human-controlled environment. A large brain with higher cognitive ability may be of higher fitness value in the wild, where food is stochastic and may be difficult to obtain at the same time as predation is a consistent risk^24^. Comparing domesticated White Leghorn laying hens with ancestral Red Junglefowl, it was recently shown that domesticated birds have higher absolute but lower relative brain weight^25^, the latter in agreement with our present findings. Furthermore, the same study used an intercross strategy to map loci associated with both body mass and brain weight, and found that the genetic architecture is not a major constraint on brain size evolution. Our results suggest that the genetic underpinning of brain size may in fact partly be associated with genes affecting fear-related behaviour.

Interestingly, the decreased brain size in low fear animals was not generally reflected in the different brain parts. Whereas low fear animals had relatively smaller telencephalon, their cerebellum were in fact relatively larger. This may indicate an adaptive modification of brain functions, in line with the mosaic hypothesis^26^. Cerebellum has many diverse functions, and is involved in, e g, movement and reflex control and in cognitive ability^27, 28^. Recently, it has been implicated to be important also in relation to social behaviour^29^. Henriksen, *et al*.^25^ reported that Red Junglefowl have smaller cerebellum than a modern domesticated egg laying chicken and also found a suggestive relationship between cerebellum size and broodiness behaviour. Our results suggest that this difference may possibly have originated already early during domestication, as a result of correlated effects of tameness. Speculatively, the increase in relative cerebellum may somehow be related to either the increased body size of these birds, or to the modifications in reproductive behaviour.

A reduced size of telencephalon has earlier been described in a number of domesticated birds such as ducks^30^, turkeys^31^ and pigeons^32^. Telencephalon is among others involved in learning, including associative learning^33^. It is possible that learning ability is reduced in domesticates as a consequence, perhaps enabled by the reduced environmental complexity of captive environment.

Sizes of heart and spleen were also affected by the selection, with a decreased size in low fear birds. Similar differences have been recorded between wild and domesticated mink^34^. In mammals, the ratio between spleen and heart sizes is very stable^35^ and Kruska and Schreiber^34^ argue that a high circulatory capacity is more important in the wild than in captivity, which again would support an adaptive organ modification during domestication. A large spleen has also been suggested to be an adaptation to high parasite pressure or a symptom on an ongoing infection^36^. Possibly, our results indicate that tamer birds experience lower demands on the immune system. In an analysis of brain gene expression differences in the same animals, many of the genes that were differentially expressed between the selection lines were related to sperm function and immunological functions^16^.

High fear males had larger testes and also more asymmetric testis sizes (left/right proportion). Asymmetric testes are common in chickens, and are typically seen in populations with a high degree of sexual selection. Speculatively, our results could therefore possibly reflect reduced sexual selection in less fearful animals. As mentioned, differentially expressed genes in the brain between the selection lines involve several sperm related transcripts^16^. Despite the differences in gene expression no skewness in actual reproductive ability of the animals in this study has been assessed. A speculative possibility would be that there is an adaptive pleiotropy between fearfulness and testis size. It could also be noted that increased testosterone levels in chickens have been associated with reduced testis size^37^ and we cannot exclude that social stress from the home pens affect testis size differently in the selection lines.

Several of the selection induced size differences in brain parts showed significant interactions between selection and sex. This suggests that selection may have affected the two sexes differently. This could possibly be due to some fear-related genes being located on sex-chromosomes, which should be studied further in future experiments.

## Conclusion

Our results show that the size of the brain and other vital organs change as a result of selection for reduced fear of humans in the present population of Red Junglefowl. This may be related to general evolutionary constraints on evolution, caused by, for example, pleiotropy and linkage of genes involved, and allowing adaptations to the “human niche” in Red Junglefowl. The modifications generally go in the direction expected from the domesticated phenotype, indicating that tameness may be a driving factor underlying organ size changes. In combination with previous results on the same selection lines, showing secondary effects of tameness on social behaviour, metabolism, reproduction and weight^4, 14, 15^ this indicates that the domesticated phenotype may be driven by increased tameness, a necessary first step in the domestication of any species.

## Materials and methods

### Ethical note

All experimental protocols were carried out in accordance with the relevant guidelines and regulations. It was approved by the Linköping Animal Ethics Committee, license no. 122–10.

### Animals and selection procedure

We studied Red Junglefowl, maintained in our research facilities and selected for five generations for divergent responses in a fear-of-human test. In this paper, we report data for the unselected and outbred parental generation (P0) and the fifth selected generation (S5). A detailed description of the breeding and selection is found in ref. 15. Briefly, the breeding started with two generations of outbreeding between two diverse zoo populations in order to maximize the genetic diversity of the animals. The phenotypic traits and behaviour of the two original populations has previously been described by Håkansson and Jensen^38^. From the second outbred generation, the parental (P0) chickens for the selection lines were hatched. In addition to the two selected lines, an unselected line was maintained based on random mating of birds from the P0 generation with intermediate scores in the fear-of-human test. Each generation was maintained at about 30–40 individuals in each selection line, and the present results are based on animals in the fifth selected generation (S5).

The fear-of human test, on which the selection was based, is described in detail in ref. 15. Briefly the animals’ fear reactions towards a human were tested at the age of 12 weeks in every generation. The animals were individually tested in an arena (measuring 100 * 300 * 200 cm, w * l * h) where a test person gradually approached the chicken during three consecutive minutes and the test ended with the person attempting to touch the chicken. The fear reaction was assessed using one-zero sampling with an interval of 10 seconds. The behavioural recordings were then transformed to an individual fear score that ranges from 1–5 where 1 indicates low fear of human and 5 indicates high fear. For more details, see Agnvall *et al*.^15^. In the following, S5 birds from the low fear selection line will be referred to as S5 low, those from the high fear line as S5 high, and the unselected birds as S5 unselected.

All animals were pedigree hatched in Marsalles incubators (37.7 °C, 55% humidity, rotation of the eggs every hour) in the experimental hatchery located at Linköping University. Immediately after hatching the animals were weighed, vaccinated against Marek’s disease and individually wing tagged. All animals were raised together until 12 weeks of age at which point they were sex separated but still kept in groups consisting of birds from all selection groups. At the age of 5 weeks the chickens were moved to floor pens (300 × 300 × 300 cm) containing shelves with nest boxes and perches in an experimental chicken house located 15 km from Linköping University. At all times, the birds had access to food and water *ad lib*. For details regarding rearing conditions, see Agnvall *et al*.^15^. From generation S4 and onwards the grown-up animals had access to an outdoor aviary (300 × 300 × 200 cm) furnished with branches and a dustbath.

### Sample collection

At the age of 48–54 weeks the animals were weighed and killed by rapid decapitation, in total 67 animals from P0 (37 females and 30 males) and 64 animals from S5 (29 females; 11 H, 9 L, 9U and 35 males; 12 H, 15 L, 8U). The brain was immediately removed and weighed as an entire unit. It was then divided into four gross parts (referred to as telencephalon, cerebellum, mid brain and optic lobes). The different parts as well as the entire brain were weighed on a balance with a precision of 0.1 g. Liver, spleen, testicles and heart were also collected and weighed.

For further analysis, both the absolute and the relative weights of each organ were used. Relative weight of body organs were calculated by dividing organ weight by total body weight, and for each of the brain parts, also the relative weight in relation to total brain weight was calculated. Furthermore, also the proportion between left and right testis weight was calculated.

### Statistical analysis

Data were first analysed within each generation. We then compared the data for the three selection lines in S5 against the average values of the same data in P0, and report the results of the post hoc comparisons performed when significant effects were detected. Mean values ± SEM were calculated for all variables. The data were checked for normality using Q-Q plots of residuals, and were in most cases found to be sufficiently normally distributed to allow usage of ANOVA for analysing effects of sex, selection and their interaction. When significant deviations from normality were detected, the data were log transformed. The diagrams all show original data values. The program Statsoft Statistica 12 was used for the analysis.

### Data accessibility

The datasets supporting this article have been uploaded as part of the electronic supplementary material.

## Electronic supplementary material

Supplementary Figure 1

Dataset 1
