# Supplementary material for: Brain size is reduced by selection for tameness in Red Junglefowl– correlated effects in vital organs
Source: Sci Rep. 2017 Jun 12;7:3306. doi: 10.1038/s41598-017-03236-4 (PMC5468340; doi:10.1038/s41598-017-03236-4)

Supplementary Fig 1

Brain size is reduced by selection for tameness in Red Junglefowl – correlated effects in vital organs

*Agnvall Beatrix, Bélteky Johan and Jensen Per**

Supplementary Fig 1. Cull weight (g), relative brain weight (% of body weight), and relative weights of cerebellum and telencephalon (% of brain weight) in males and females from the fifth selected generation of Red Junglefowl (S5).

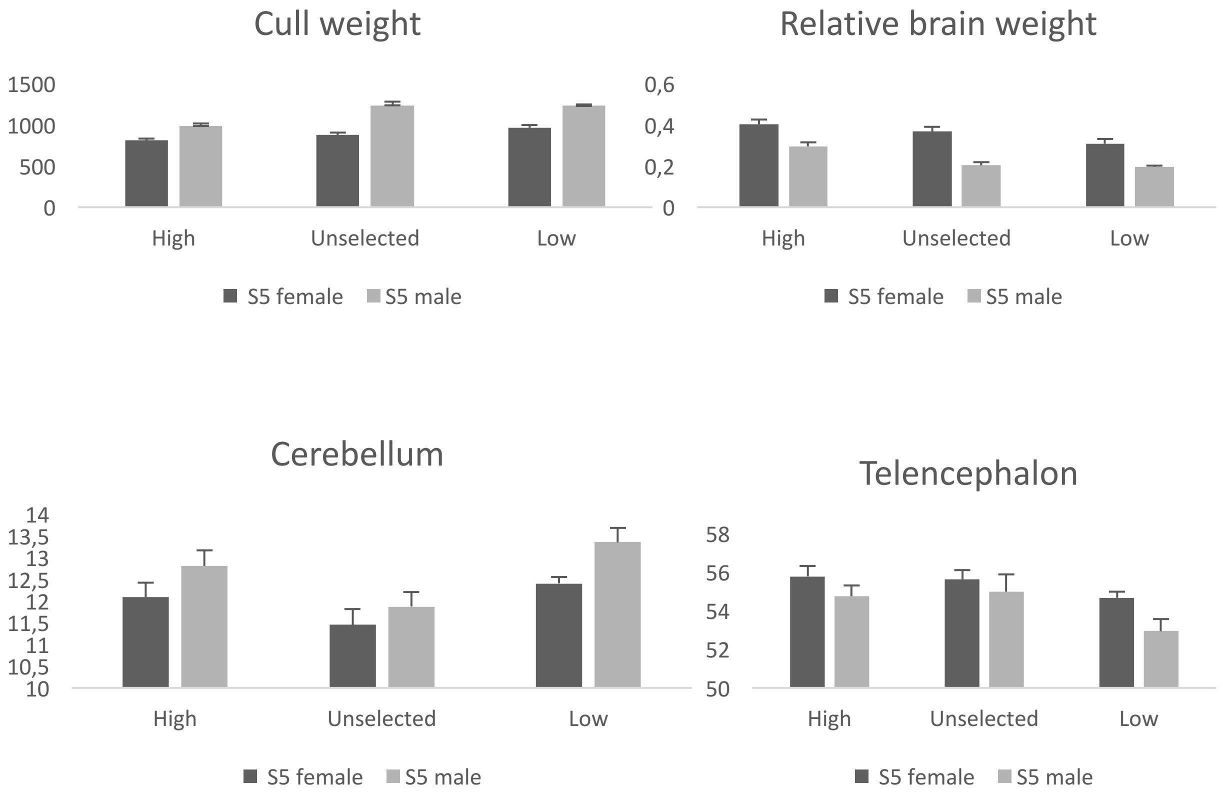

Supplement: Supplementary file 1 — Supplementary Figure 1 [file 41598_2017_3236_MOESM1_ESM.pdf]
